# Case Report of a Man with Right Eye Pain and Double Vision

**DOI:** 10.21980/J8KW7G

**Published:** 2022-01-15

**Authors:** Nicolas Kahl, Maria Pelucio

**Affiliations:** * University of California, San Diego, Department of Emergency Medicine, San Diego, CA

## Abstract

A 39-year-old previously healthy male presented with three days of right eye pressure and one day of binocular diplopia. He denied history of trauma, headache, or other neurological complaints. He had normal visual acuity, normal intraocular pressure, intact convergence, and no afferent pupillary defect. His neurologic examination was non-focal except for an inability to adduct the right eye past midline on cranial nerve examination, with left-beating nystagmus noted in the left eye. MRI brain was obtained, showing right cavernous sinus lateral wall contrast enhancement. No other evidence of neoplasm, ischemia, infection, or aneurysm was found. The patient’s deficits rapidly improved with corticosteroids. This is the hallmark of Tolosa-Hunt syndrome (THS), characterized as idiopathic inflammation of the cavernous sinus. The patient was discharged on a prednisone taper, with continued improvement at outpatient neurology follow-up. Tolosa-Hunt syndrome is one of the rare disorders recognized by the National Organization for Rare Disorders (NORD), and has an estimated incidence of one case per million per year.

**Topics:**

Tolosa-Hunt syndrome, cranial nerve deficits, diplopia, neurologic examination.

[Fig f1-jetem-7-1-v22] [Fig f2-jetem-7-1-v22] [Fig f3-jetem-7-1-v22]

**Figure f1-jetem-7-1-v22:**
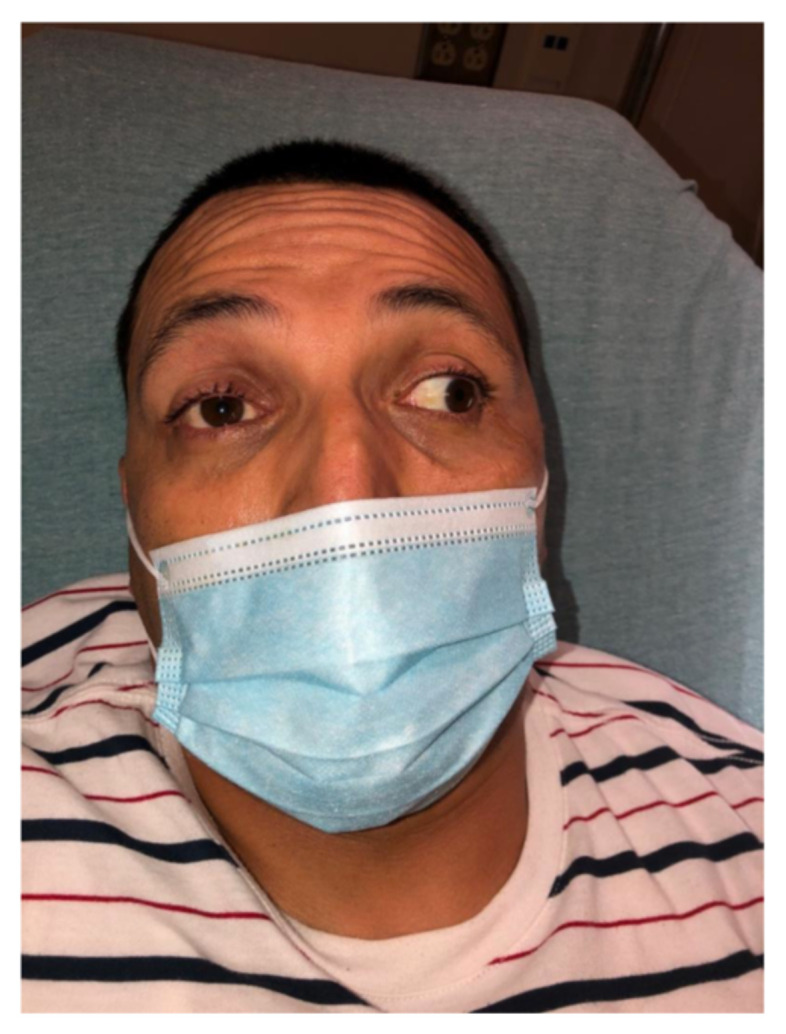


**Figure f2-jetem-7-1-v22:**
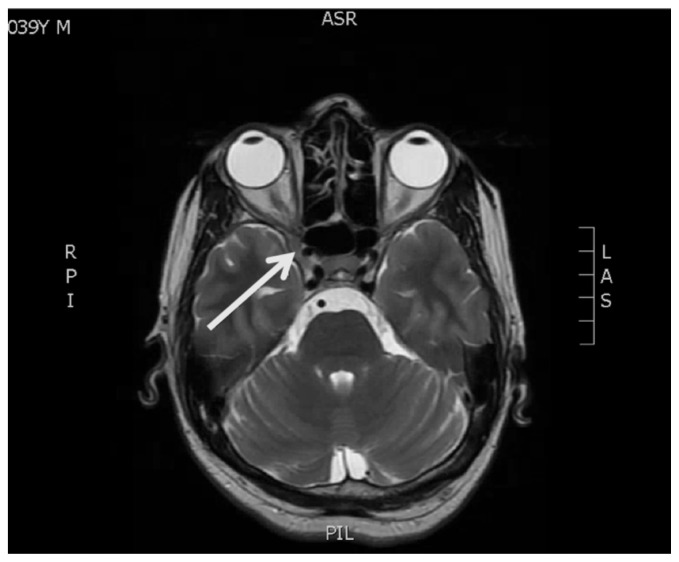


**Figure f3-jetem-7-1-v22:**
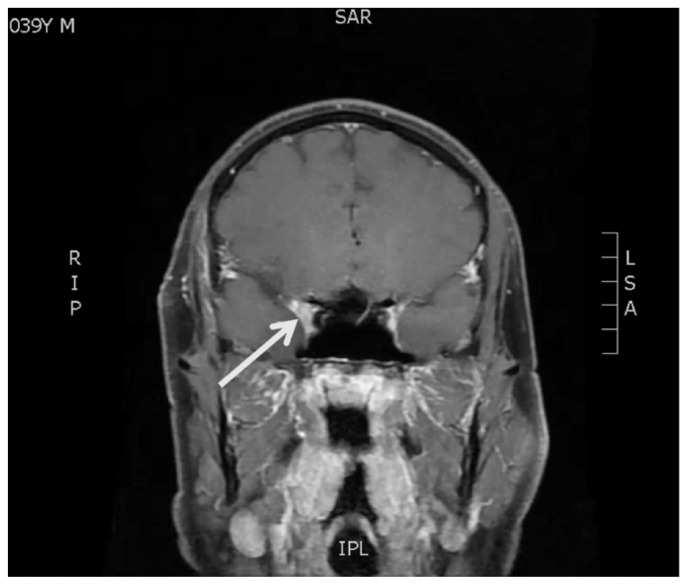


## Brief introduction

Performing a careful physical exam is essential to the evaluation of patients with visual complaints in the emergency department. The differential diagnosis for cranial nerve deficit is broad, always requiring a thorough neurologic exam, and often neurologic imaging or neurology consult. Classic emergent diagnoses associated with cranial nerve deficits are posterior communicating artery aneurysm, septic cavernous sinus thrombosis, internal carotid artery aneurysm, invasive fungal sinusitis, and lymphoma among others.^1^–^5^ This is a case of a rare cause of cavernous sinus syndrome and cranial nerve deficit with an estimated incidence of one case per million per year. Written patient consent was obtained for inclusion of photographs and images accompanying this article.

## Presenting concerns and clinical findings

A 39-year-old male presents to the emergency department with right eye pressure for three days, now with binocular diplopia since yesterday morning that is improved when he closes one eye. He denies headache but reports some nausea and dizziness secondary to his diplopia. He denies fever, foreign body sensation, discharge from his eye, wearing glasses, contact use, history of similar episodes in the past, difficulty walking or other neurological deficit. He has not had recent trauma, recent travel, weight loss, abdominal pain, dysphagia, dysarthria, dyspnea, weakness, vertigo, numbness, fatigue, memory dysfunction, or other complaints. He denies history of tuberculosis (TB), syphilis, or sexually transmitted infections. He has no family history of brain tumor, aneurysm, hemorrhage, or seizures. The patient was born in Mexico, but has not left the United States in over ten years. He works in construction and lives with his wife and cat. He drinks two to three beers per day and smokes five cigarettes per day. He denies cocaine, amphetamine, or intravenous drug use. Occasionally he uses edible cannabinoid, but none in the past week. His vaccinations are up to date. No known COVID-19 exposure.

## Significant findings

Paralysis on adduction of the right eye and horizontal nystagmus in the left eye on left lateral gaze. Pupils are equal, round, reactive to light, and the remainder of the patient’s neurologic examination did not have any focal findings. This patient demonstrated a cranial nerve III palsy, necessitating thorough neurologic exam and imaging.

## Patient course

In this case, MRI brain and orbits showed T2 hypointense signal and diffuse contrast enhancement along the lateral wall of the right cavernous sinus. The patient subsequently had MRI angiography, digital subtraction angiography, and lumbar puncture without evidence of neoplasm, ischemia, infection, or aneurysm. There were no significant barriers to diagnostic tests because the patient presented to a quaternary care hospital. In addition to adduction deficits, the patient progressed to have abducens nerve deficits as an inpatient: both deficits improved rapidly with corticosteroids. This is the hallmark of Tolosa-Hunt syndrome, characterized as idiopathic inflammation of the cavernous sinus. The patient was treated with prednisone 80 mg by mouth daily as an inpatient for three days. He was discharged on a prednisone taper with a decrease in dose every two weeks: 60mg, 40mg, 20mg and finally 10mg, with continued improvement at outpatient neurology follow-up. The patient adhered to the recommended treatment regimen and reported minimal side effects at follow-up neurology appointments.

## Discussion

The following criteria define Tolosa-Hunt syndrome: unilateral eye pain, associated palsies of cranial nerves III, IV, and/or VI, and relief of symptoms within 48 hours after administration of steroids. MRI or biopsy-proven granulomatous inflammation of the cavernous sinus, superior orbital fissure, or orbit is also integral to diagnosis.^6^ The average age of onset is 41 years old, but pediatric cases have been observed.^7^ The pathogenesis is believed to be caused by inflammatory cells such as lymphocytes and plasma cells exerting pressure on the cranial nerves within the tight space of the cavernous sinus. No clear etiology has been established, but it has been observed in association with other autoimmune disorders such as systemic lupus erythematosus and sarcoidosis.^8^ Cavernous sinus lymphoma has been observed to closely mimic THS and should be strongly considered in the differential.^9^ Despite treatment with steroids, approximately half of patients with THS can have recurrence of symptoms and rarely suffer long term cranial nerve deficits. The literature on long term outcomes for THS patients is scarce, with recent case series noting that steroids followed by a course of immunosuppression may reduce the risk of relapse.^10^ Tolosa-Hunt syndrome is one of the rare disorders recognized by the National Organization for Rare Disorders (NORD), and has an estimated incidence of one case per million per year.^11^ The strengths of this case are the opportunity to review a classic presentation of a rare diagnosis, high quality images of physical exam and diagnostic imaging findings that support the diagnosis, and a demonstrated response of the diagnosis to its recommended initial treatment. Limitations include the challenge in finding large research studies on THS given its rarity, and the lack of a known etiology for the condition. Further study is needed to elucidate the Tolosa-Hunt syndrome, but it is important to consider in the emergency department due to significant overlap of its presentation with life-threatening causes of cavernous sinus syndrome.

## Supplementary Information






