# Supplementary figures and images for: Case Report of a Man with Right Eye Pain and Double Vision
Source: J Educ Teach Emerg Med. 2022 Jan 15;7(1):V22–5. doi: 10.21980/J8KW7G (PMC10358874; doi:10.21980/J8KW7G)

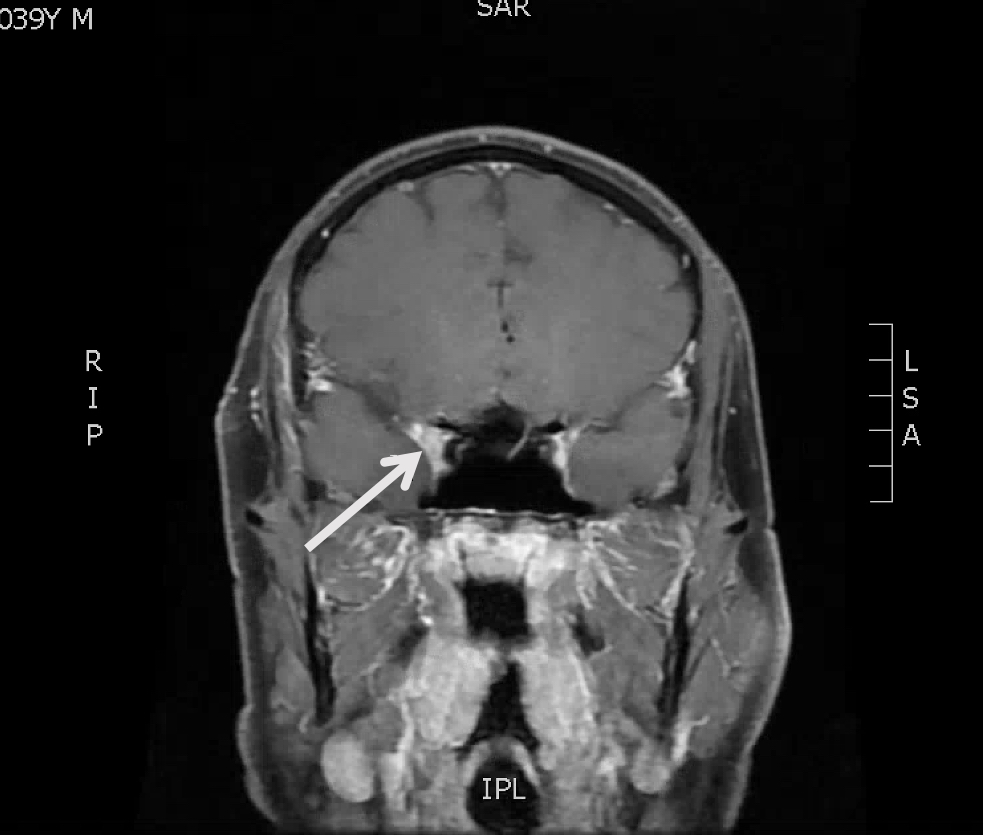

Supplement: Supplementary file 1 [file JETem-7-1-V22-supp1.jpg]

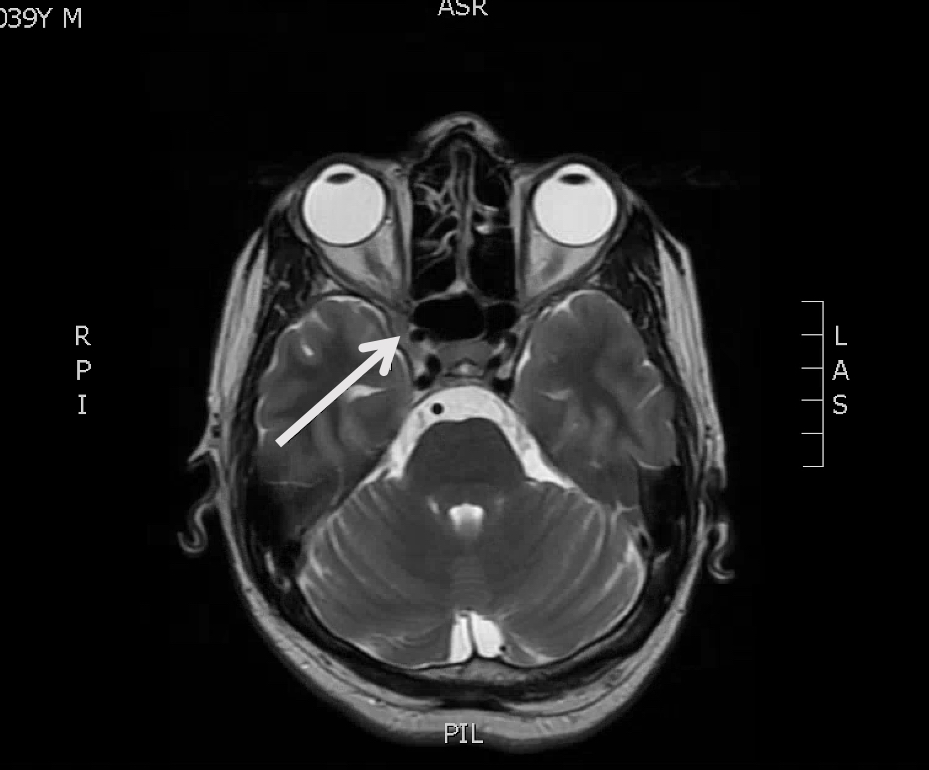

Supplement: Supplementary file 2 [file JETem-7-1-V22-supp2.jpg]

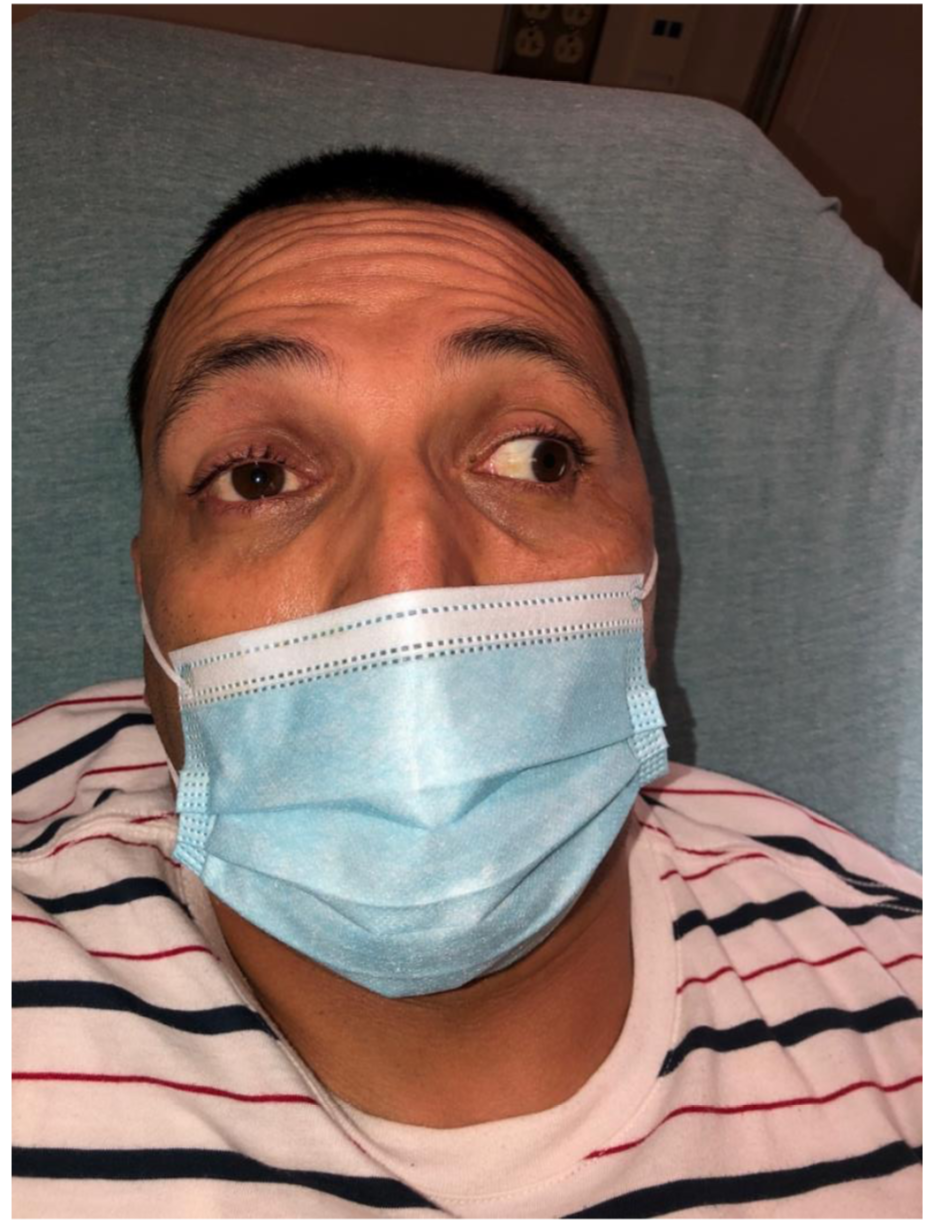

Supplement: Supplementary file 3 [file JETem-7-1-V22-supp3.jpg]
